# Supplementary material for: Epidermal chloroplasts are defense-related motile organelles equipped with plant immune components
Source: Nat Commun. 2021 May 20;12:2739. doi: 10.1038/s41467-021-22977-5 (PMC8137707; doi:10.1038/s41467-021-22977-5)
Supplement: Supplementary file 1 — Supplementary Information [file 41467_2021_22977_MOESM1_ESM.pdf]

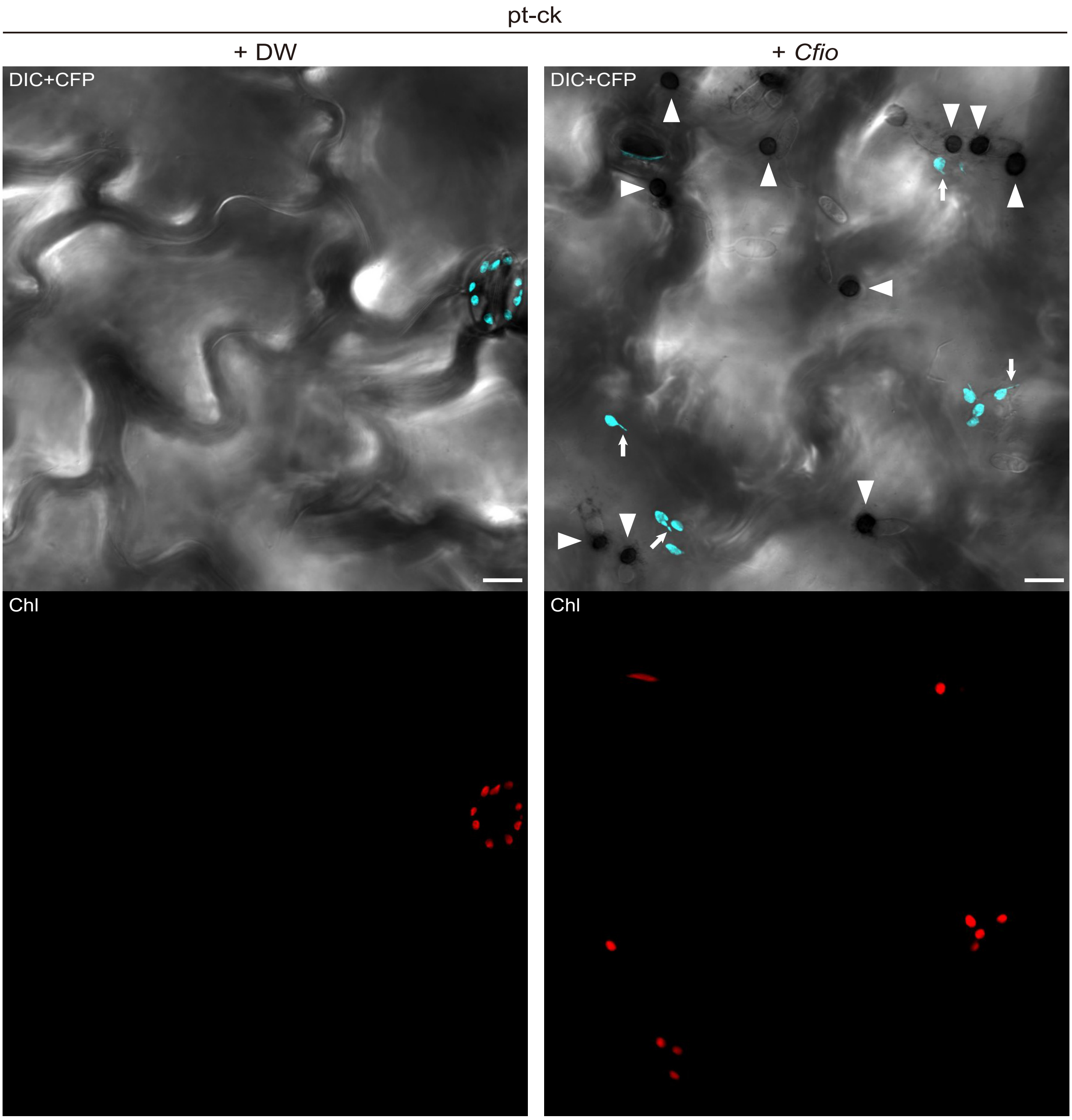

Supplementary Figure 1. Visualization of epidermal chloroplasts and stromules.

Visualization of epidermal chloroplasts and stromules with plastid-CFP marker. *Cfio* was inoculated into the cotyledons of the transgenic plant line pt-ck expressing the plastid-CFP marker, and the surface of the epidermis was examined. DW was used as a control. The epidermal chloroplasts were simultaneously visualized using chlorophyll autofluorescence. The DIC image was captured by confocal microscopy at 2 dpi. Arrowheads and arrows indicate melanized appressoria and stromules, respectively. Scale bar, 20 µm. Similar results were obtained from two independent experiments.

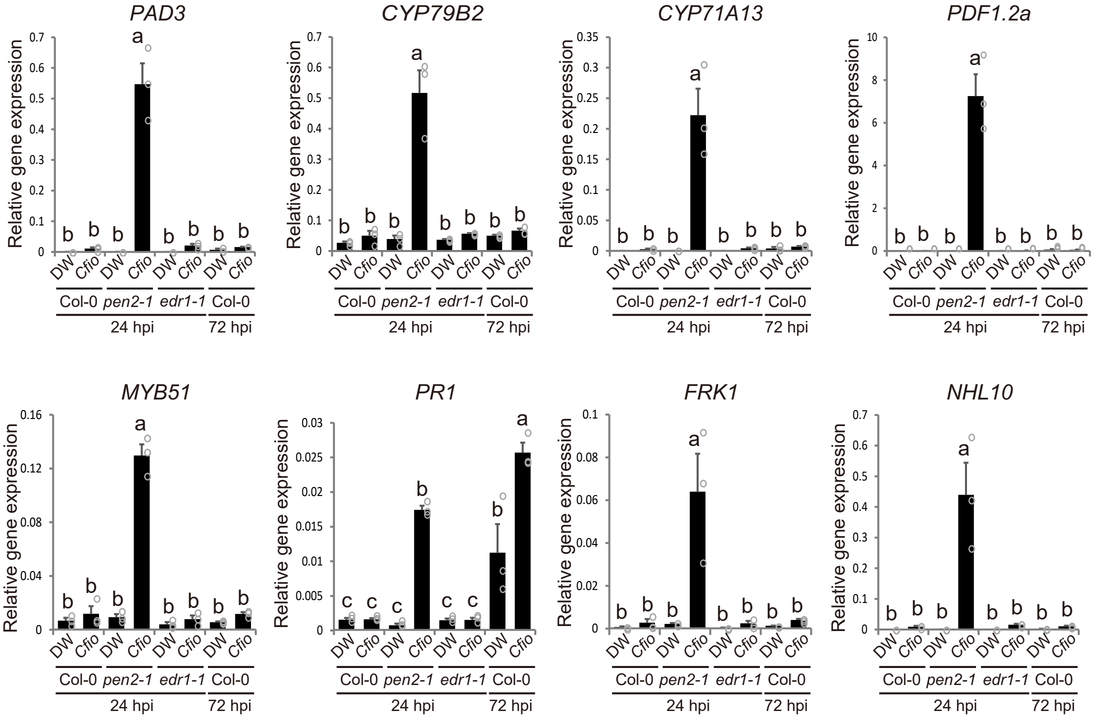

Supplementary Figure 2. Induction of defense-related gene expression in *Arabidopsis* by inoculation of *Cfio*.

Induced gene expression of *PAD3*, *CYP79B2*, *CYP71A13*, *MYB51*, *PDF1.2a*, *PR1*, *FRK1*, and *NHL10* by inoculation with *Cfio*. Cotyledons of the indicated *Arabidopsis* were inoculated with *Cfio*, and gene expression was investigated at 24 and 72 hpi by RT-qPCR. DW was used as a control. Means and SE were calculated from three independent experiments. Means not sharing the same letter are significantly different (P < 0.05, two-way ANOVA with Tukey's HSD test).

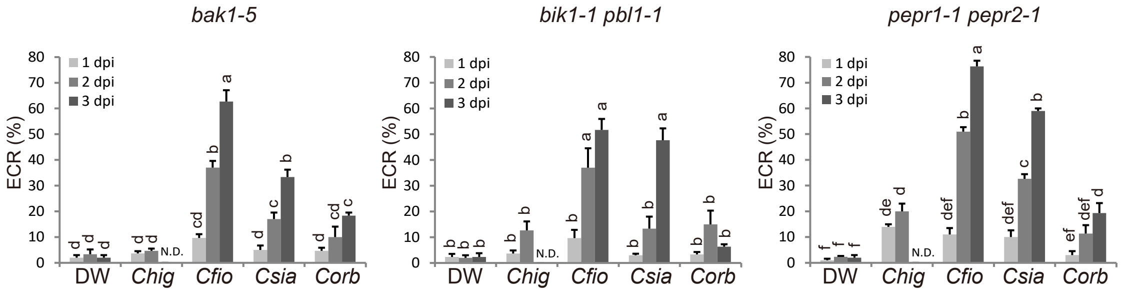

Supplementary Figure 3. Normal ECR of *pepr1 pepr2*, *bak1-5*, and *bik1 pbl1* mutants against *Colletotrichum* fungi.

ECR of *pepr1-1 pepr2-1*, *bak1-5*, and *bik1-1 pbl1-1* after *Colletotrichum* inoculation. The ratio of epidermal cells with surface chloroplasts was investigated at 1, 2 and 3 dpi. A total of 100 cells in contact with the melanized appressorium were observed. The mean and SE values were calculated for the three independent plants. DW was used as a control. N.D.: not determined due to damage to epidermal cells by fungal invasion. Means not sharing the same letter are significantly different ($P < 0.05$, two-way ANOVA with Tukey's HSD test).

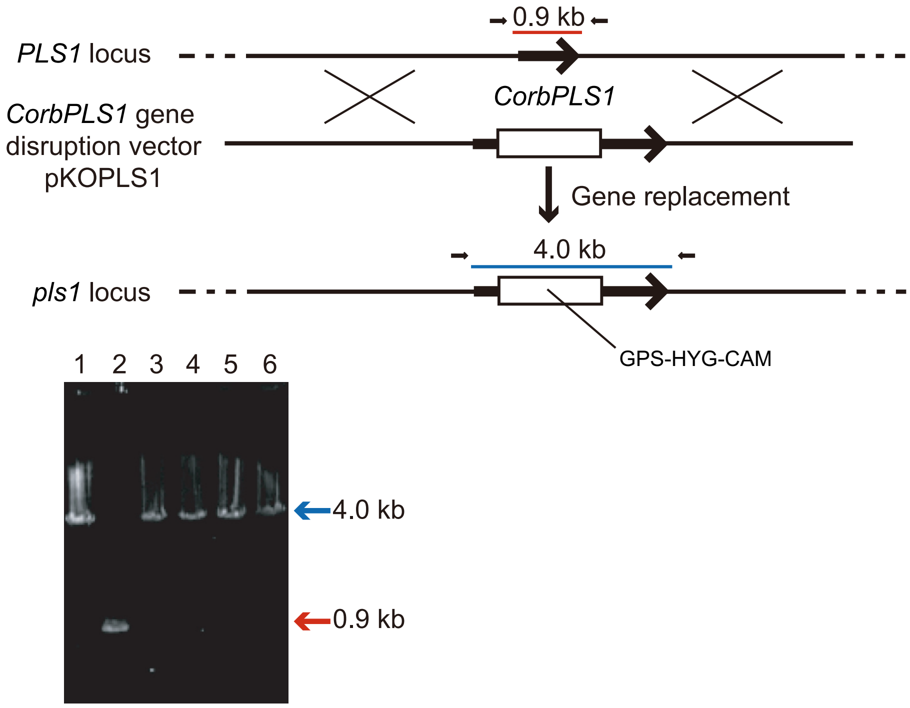

Supplementary Figure 4. The generation of *PLS1* gene disruption mutants in *Corb*.

Disruption of the *PLS1* gene in *Corb*. The *PLS1* locus of *Corb* and the *CorbPLS1* gene disruption vector pKOPLS1, containing a transposon carrying the hygromycin resistance gene and chloramphenicol resistance gene (GPS-HYG-CAM cassette), was inserted into the *PLS1* locus. Genomic PCR analysis was performed using DNA isolated from the wild-type strain 104-T or strains transformed with pKOPLS1. The *PLS1* locus was amplified from the *PLS1* cosmid (lane 1), genomic DNA from the wild-type strain 104-T (lane 2), and four *pls1* transformants (lanes 3–6). The primers used for the genomic PCR are indicated by arrows.

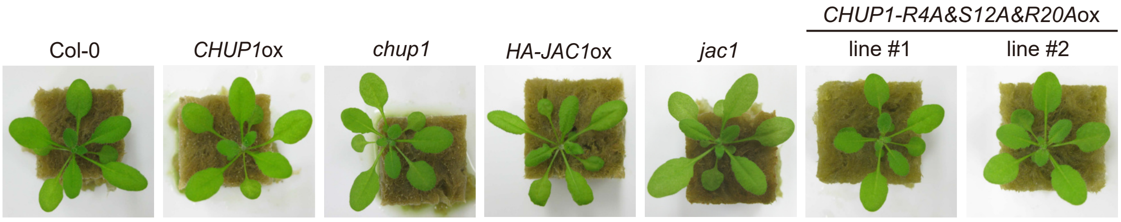

Supplementary Figure 5. Normal morphology of *CHUP1*ox, *CHUP1-R4A&S12A&R20A*ox, *chup1*, and *jac1* plants.

Morphological phenotypes of Col-0, *CHUP1*ox, *chup1*, *HA-JAC1*ox, *jac1*, and *CHUP1-R4A&S12A&R20A*ox plants. Photos were taken from 25-day-old plants.

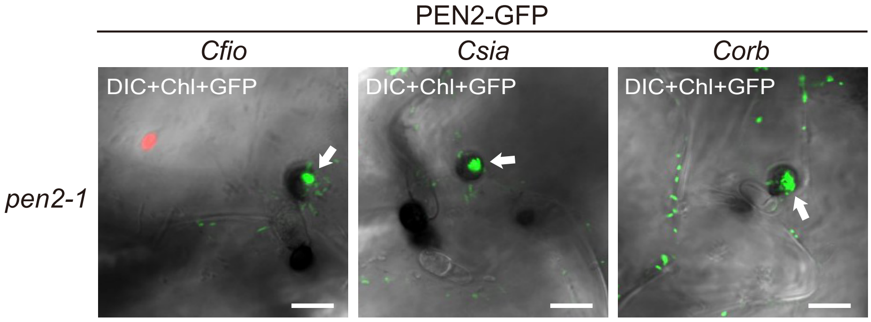

Supplementary Figure 6. Focal accumulation of PEN2 at entry trial sites of *Colletotrichum* fungi.

Focal accumulation of PEN2 at the fungal entry trial sites. *Cfio*, *Csia*, and *Corb* were inoculated onto the transgenic *pen2-1* plant expressing the PEN2-GFP fusion protein[3]. Inoculated plants were incubated for 2 days, and DIC images were captured by confocal microscopy. Epidermal chloroplasts were visualized using chlorophyll autofluorescence. The arrows indicate PEN2-GFP fusion proteins localized at the entry trial sites of *Colletotrichum* fungi. Scale bar, 10 µm. Similar results were obtained from two independent experiments.

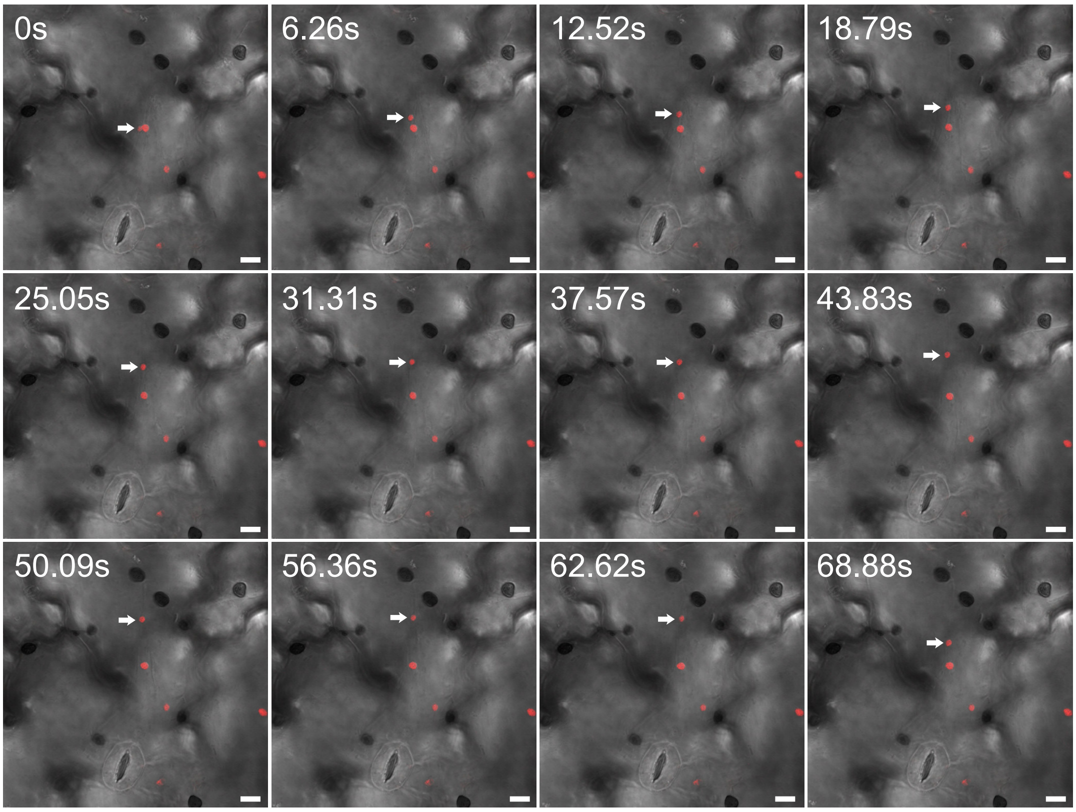

Supplementary Figure 7. The intracellular movement of chloroplast in ECR-activated epidermal cell.

Intracellular movement of chloroplast in ECR-activated epidermal cell. Time-lapse images of the surface of epidermal cells after *Corb* inoculation. *Corb* was inoculated onto *pen2-1* plants, and ECR was observed, and DIC images were captured at 3 dpi. Epidermal chloroplasts were visualized by chlorophyll autofluorescence. The arrows indicate a moving chloroplast. Scale bar, 10 μm. Related to Supplementary Movie 1. Similar results were obtained from three independent experiments.

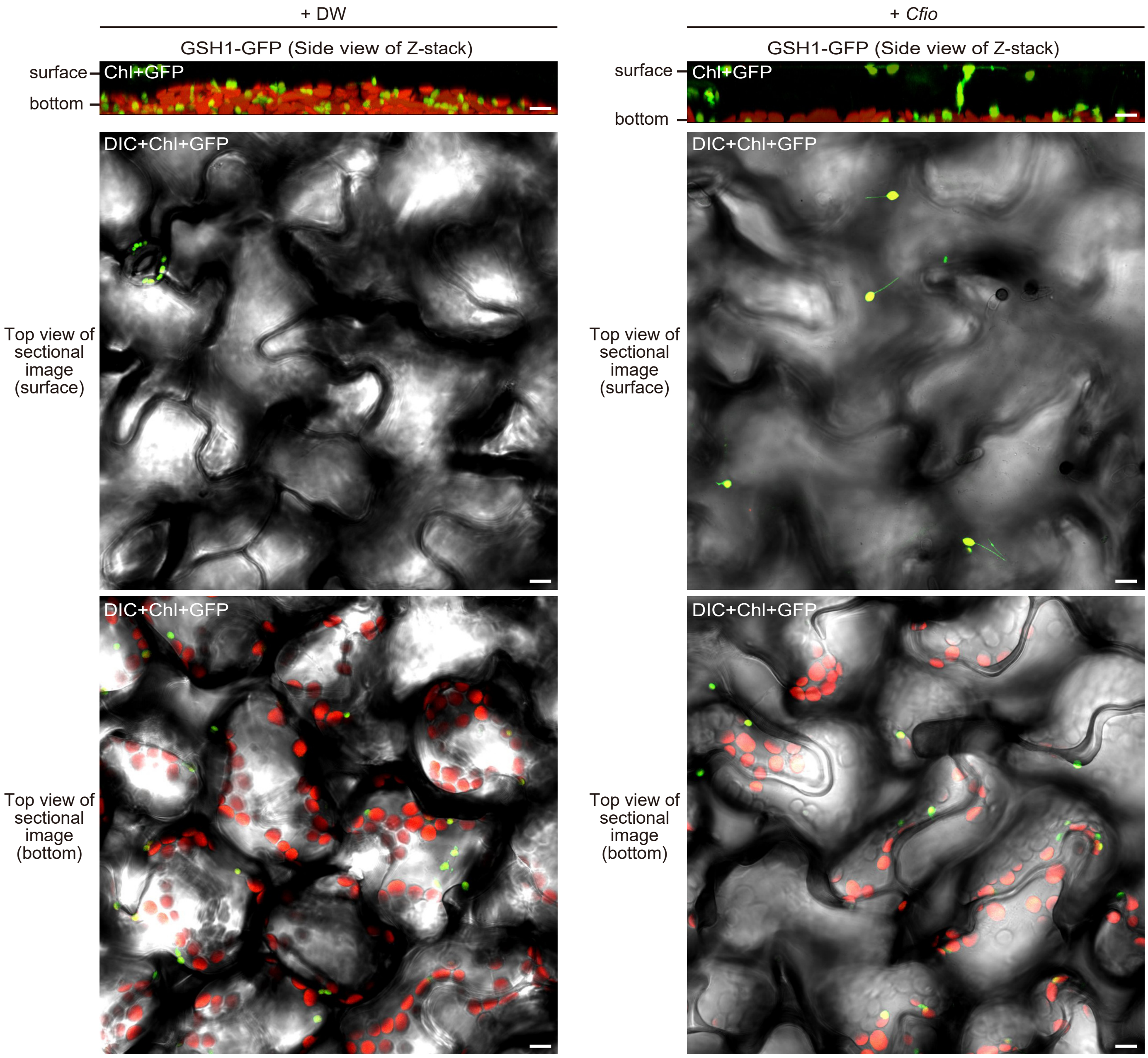

Supplementary Figure 8. Z-stack analyses of epidermal and mesophyll chloroplasts in plants expressing fluorescently-labeled immune components.

Side view of Z-stack images of the *gsh1-1* plants expressing *GSH1*::GSH1-GFP, Col-0 plants expressing *CaMV 35S*::EDS5-sfGFP, and Col-0 plants expressing *CaMV 35S*::CAS-sfGFP with or without fungal inoculation (upper). The epidermal surface (middle) and bottom (lower) sectional images were extracted and their top views were displayed. Scale bar, 10 µm. Similar results were obtained from three independent experiments.

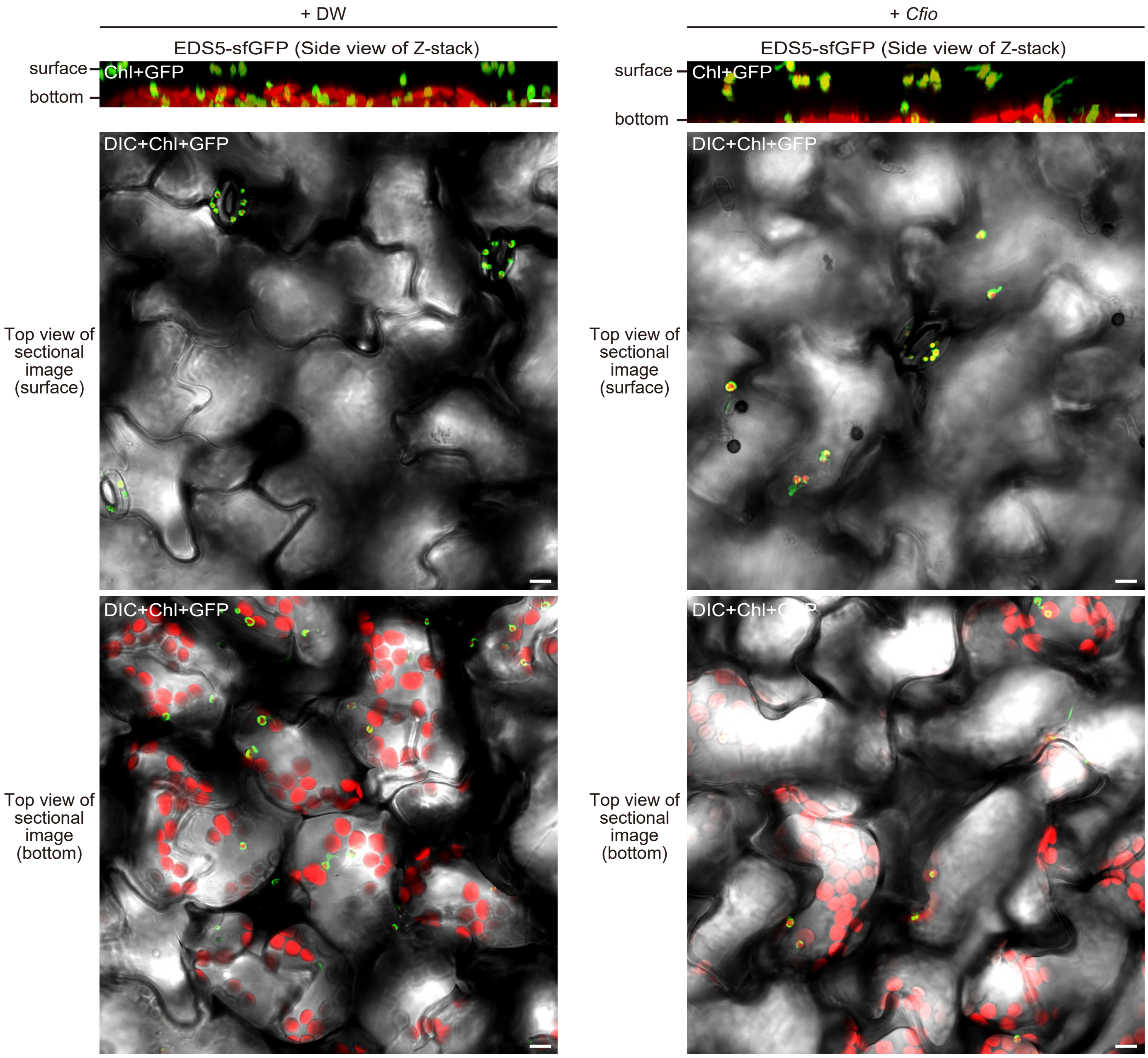

Supplementary Figure 8. Z-stack analyses of epidermal and mesophyll chloroplasts in plants expressing fluorescently-labeled immune components.

Side view of Z-stack images of the *gsh1-1* plants expressing *GSH1*::GSH1-GFP, Col-0 plants expressing *CaMV 35S*::EDS5-sfGFP, and Col-0 plants expressing *CaMV 35S*::CAS-sfGFP with or without fungal inoculation (upper). The epidermal surface (middle) and bottom (lower) sectional images were extracted and their top views were displayed. Scale bar, 10 µm. Similar results were obtained from three independent experiments.

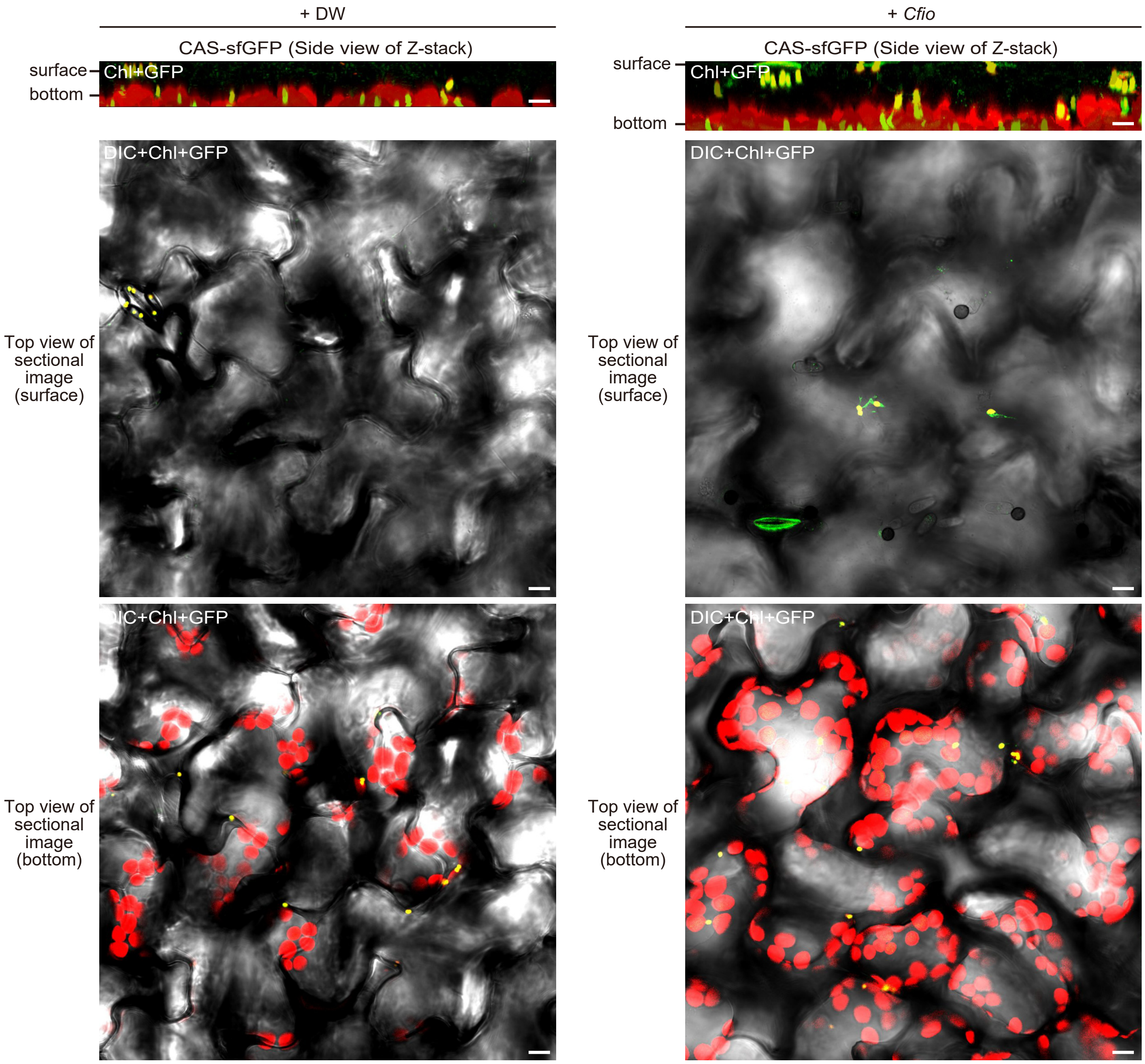

Supplementary Figure 8. Z-stack analyses of epidermal and mesophyll chloroplasts in plants expressing fluorescently-labeled immune components.

Side view of Z-stack images of the *gsh1-1* plants expressing *GSH1*::GSH1-GFP, Col-0 plants expressing *CaMV 35S*::EDS5-sfGFP, and Col-0 plants expressing *CaMV 35S*::CAS-sfGFP with or without fungal inoculation (upper). The epidermal surface (middle) and bottom (lower) sectional images were extracted and their top views were displayed. Scale bar, 10 µm. Similar results were obtained from three independent experiments.

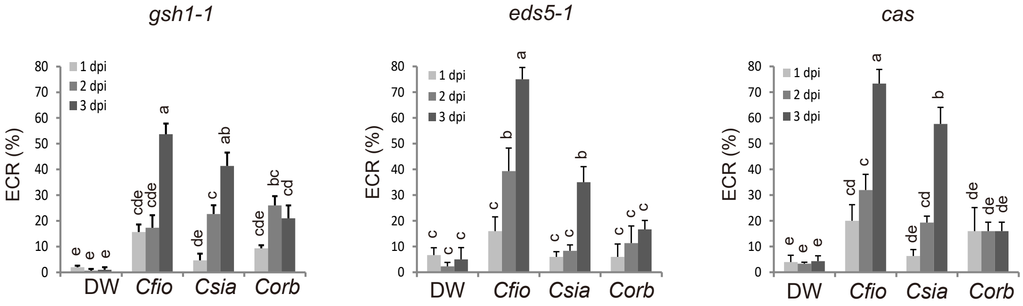

Supplementary Figure 9. Normal ECR of *gsh1*, *eds5*, and *cas* mutants.

ECR of *gsh1-1*, *eds5-1*, and *cas* mutants after nonadapted *Colletotrichum* inoculation. *Cfio*, *Csia*, and *Corb* were inoculated onto the indicated plants, and the ratio of epidermal cells with surface chloroplasts was investigated at 1, 2, and 3 dpi. A total of 100 cells that were in contact with the melanized appressorium were observed. DW was used as a control. The mean and SE values were calculated from three independent plants. Means not sharing the same letter are significantly different ($P < 0.05$, two-way ANOVA with Tukey's HSD test).

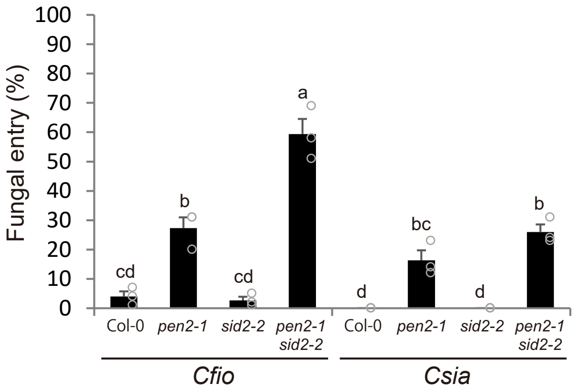

Supplementary Figure 10. The effects of *sid2* mutation on preinvasive defense against *Cfio* and *Csia*.

Effect of the *sid2-2* mutation on preinvasive NHR against *Colletotrichum* fungi. Conidia of *Cfio* and *Csia* were inoculated onto the cotyledons of the indicated *Arabidopsis* plants. The entry rate of each fungus was quantified at 4 dpi. In total, 100 melanized appressoria were investigated. The mean and SE values were calculated from three independent plants. Means not sharing the same letter are significantly different ($P < 0.05$, two-way ANOVA with Tukey's HSD test).

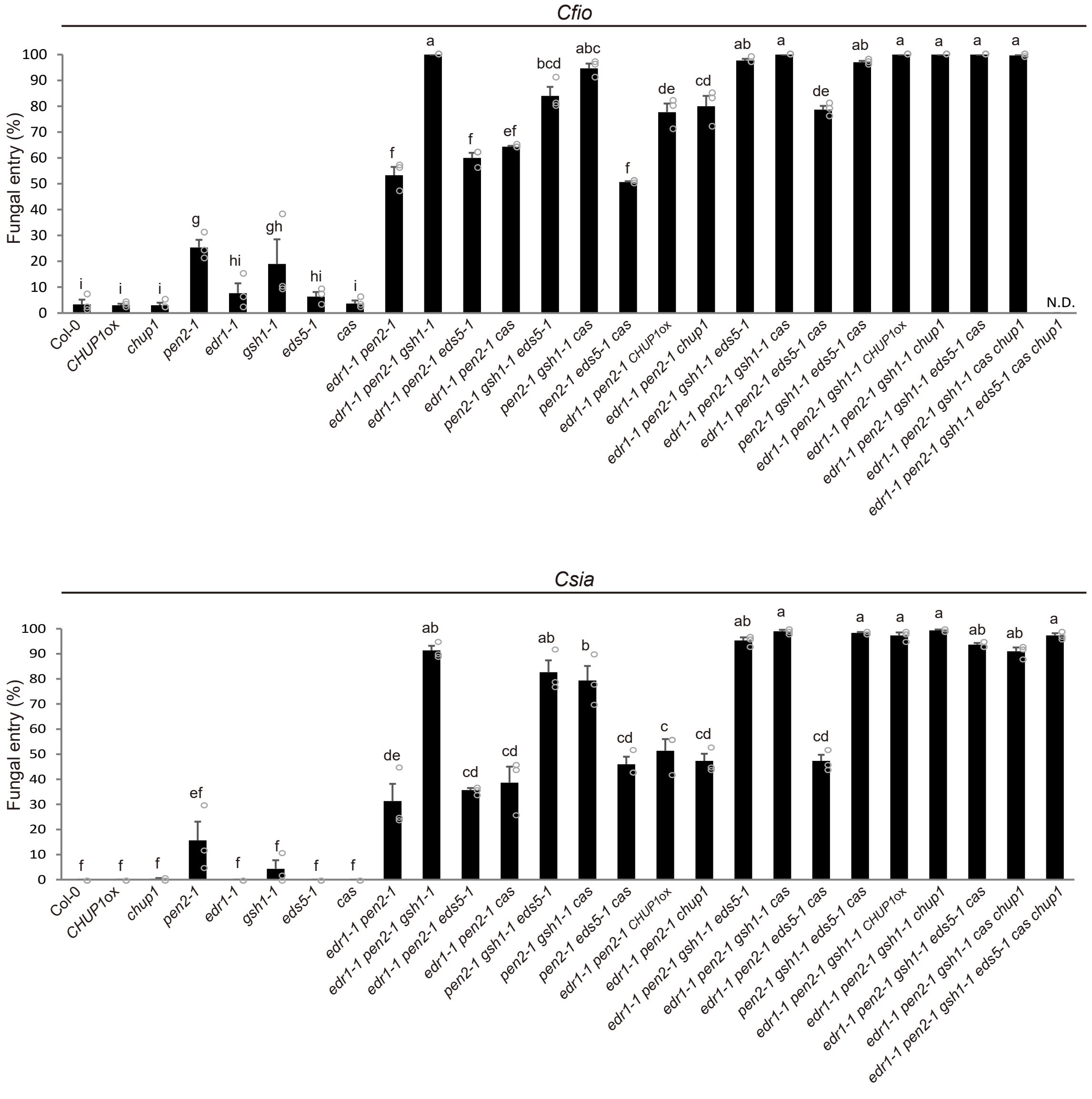

Supplementary Figure 11. The effects of multiple mutations on preinvasive NHR against *Cfio* and *Csia*.

Invasion of *Cfio* and *Csia* in the epidermis of *Arabidopsis* with multiple mutations. *Cfio* and *Csia* were inoculated onto the cotyledons of the indicated plants. Entry rate was quantified at 4 dpi. In total, 100 melanized appressoria were investigated. The mean and SE values were calculated for the three independent plants. N.D.: not determined due to the collapse of inoculated cotyledons by fungal infection. Means not sharing the same letter are significantly different ($P < 0.05$, one-way ANOVA with Tukey's HSD test).

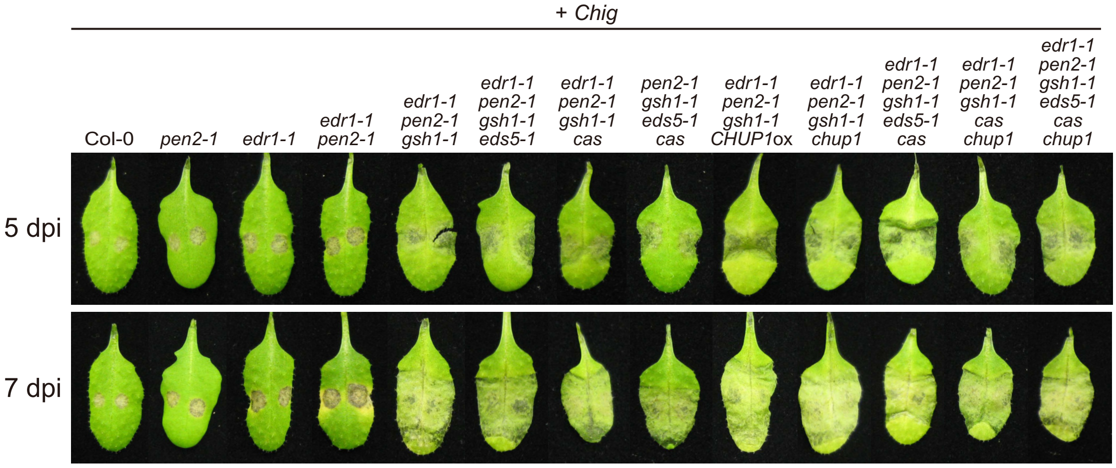

Supplementary Figure 12. The effects of multiple mutations on *Arabidopsis* resistance against *Chig*.

Pathogenicity of *Chig* in multiple *Arabidopsis* mutants. A conidial suspension of adapted *Chig* was inoculated onto the leaves of the indicated plants and incubated for 5 and 7 days.

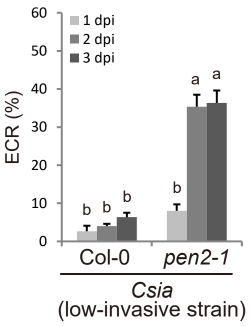

Supplementary Figure 13. ***Csia*** **(low-invasive strain)-induced ECR occurs not in wild-type, but does occur in the** ***pen2*** **mutant.**

The ratio of epidermal cells with surface chloroplasts was investigated at 1, 2, and 3 dpi. A total of 100 cells in contact with the melanized appressorium were observed. The mean and SE values were calculated for the three independent plants. Means not sharing the same letter are significantly different ($P < 0.05$, two-way ANOVA with Tukey's HSD test).

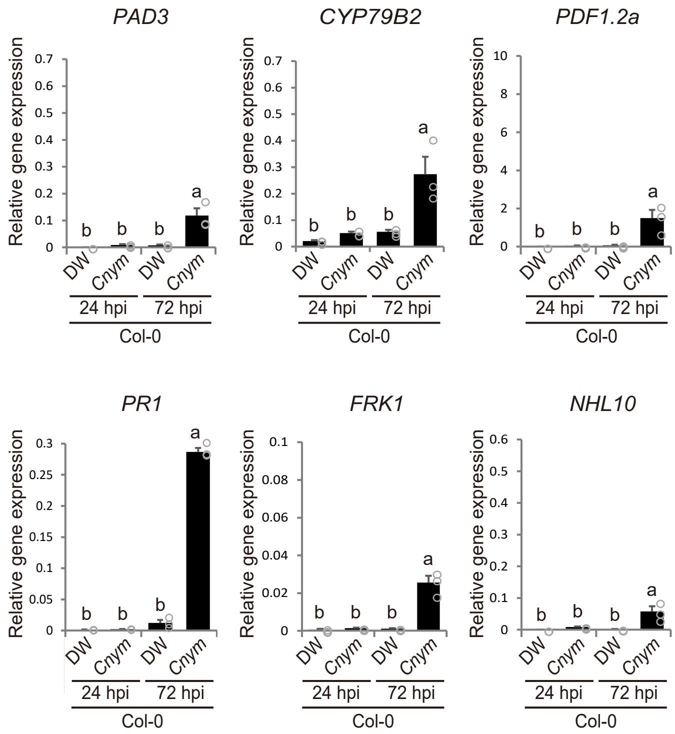

Supplementary Figure 14. Induction of defense-related gene expression in *Arabidopsis* by inoculation of *Cnym*.

Induced gene expression of *PAD3*, *CYP79B2*, *PDF1.2a*, *PR1*, *FRK1*, and *NHL10* by inoculation with *Cnym*. Cotyledons of Col-0 were inoculated with *Cnym*, and gene expression was investigated at 24 h and 72 hpi by RT-qPCR. DW was used as a control. Means and SE were calculated from three independent experiments. Means not sharing the same letter are significantly different ($P < 0.05$, two-way ANOVA with Tukey's HSD test).

Col-0 + *P. syringae* pv. *tomato* DC3000 (*Pst* DC3000)

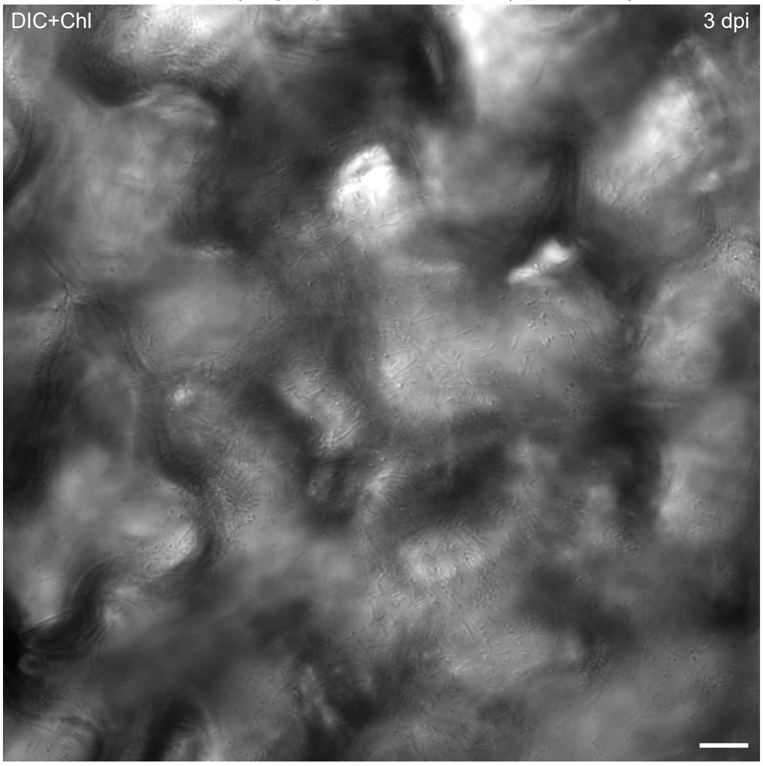

Supplementary Figure 15. Apoplastic bacterial pathogen does not induce ECR in *Arabidopsis*.

ECR was not activated after inoculation with *Pseudomonas syringae* pv. *Tomato* (*Pst*) DC3000. Cotyledons of Col-0 were drop-inoculated with *Pst* DC3000, and the surface chloroplasts were investigated by visualizing chlorophyll autofluorescence at 3 dpi. Similar results were obtained from two independent experiments.

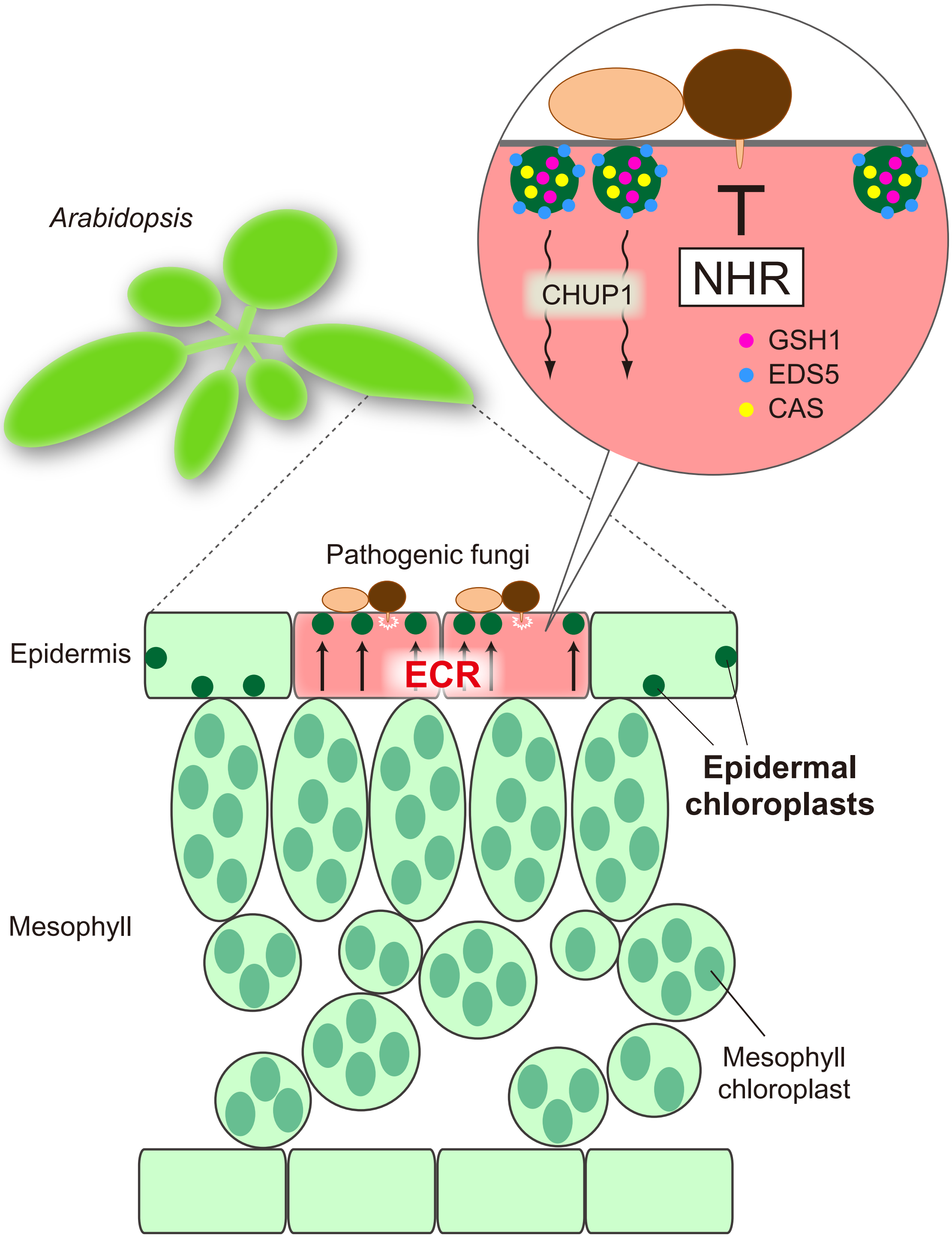

Supplementary Figure 16. Schematic overview of the ECR-related preinvasive NHR in *Arabidopsis* epidermis.

An appressorium-mediated entry trial of nonadapted fungi triggers the CHUP1-related ECR in *Arabidopsis*. The epidermal chloroplast-localized immune components GSH1, EDS5, and CAS also dramatically changed their intracellular locations by ECR. The ECR and these immune components contribute to epidermal preinvasive NHR against fungal pathogens.

Supplementary Table 1. Primers used for the plasmid construction.

**Name**	**Sequence (5’-3’)**	**Plasmid**
AtCHUP1-NdeI-f	GCCATATGTTTGTCCGGATAGGG	pRI101-AtCHUP1
AtCHUP1-R4A&S12A&R20A -NdeI-f	GCCATATGTTTGTCGCGATAGGGTTTGTTGTTGCTGCTGCCATTGCAGCAGTTACTGTTAAGGCGCTCAACG	pRI101-AtCHUP1-R4A&S12A&R20A
AtCHUP1pro-PstI-f	CATTGGTTCTGCAGAATCATTGGCTTCGAACTTATTCTCACAC	pRI-AtCHUP1p-AtCHUP1
AtCHUP1-SmaI-r	TCCCCCGGGTCAGTTTACAGATTCTTCTTC	pRI101-AtCHUP1, pRI101-AtCHUP1-R4A&S12A&R20A, pRI-AtCHUP1p-AtCHUP1
HA-AtJAC1-NdeI-f	GCCATATGTACCCATACGATGTTCCAGATTACGCTATGCAGACATTACCAAGCTC	pRI101-HA-AtJAC1
AtJAC1-EcoRI-r	GGAATTCTTAAACCGGTCCGAGAGTGTTG	
sfGFP-BamHI-f	CGGGATCCATGAGTAAAGGAGAAGAAC	pRI101-sfGFP-C
sfGFP-EcoRI-r	GGAATTCTTATTTGTATAGTTCATCCATGCC	
AtEDS5-SmaI-f	TCCCCCGGGATGCTAATCAAATCCCAAAGATTG	pRI101-AtEDS5-sfGFP
AtEDS5-KpnI-r	GGGGTACCAATGGATTTAATCTTCTCCACCGTG	
AtCAS-NdeI-f	GCCATATGGCTATGGCGGAAATGGC	pRI101-AtCAS-sfGFP
AtCAS-SmaI-r	TCCCCCGGGGTCGGAGCTAGGAAGGAACTTG	
DCPLS1s	ACGCCGGCCGTCATAACACCTGAATCTG	pKOPLS1
DCPLS1as	TTCAAGTTGGTAAGCGGTCGTACCACC	

Supplementary Table 2. Primers used for the genotyping of *Arabidopsis*.

Name	Sequence (5'-3')	Mutation and restriction enzyme
pen2-1F	TCAGGTAAATCAGTTCGAATCAAGAAC	*pen2-1* BamHI
pen2-1R	TGAGGAAACCTGTTGGAGAAAGGATC	
edr1-1F	CAGAGGCTGAAAGGACAGATTCTTGGTA	*edr1-1* KpnI
edr1-1R	CCTCACTGTTCTGATTGTAAGG	
gsh1-1F	TTACAACCTGTGAGAGCTCG	*gsh1-1* SphI
gsh1-1R	CTGAATCTAGATACCTTCGCATG	
eds5-1F	CTTGGTCTAATCTGATTCTTGATATGTTTTCTA	*eds5-1* XbaI
eds5-1R	GAGACTTATTCAGCTGCTTGCTTCTC	
sid2-2F	TGCAGCTTCAATGCTTCATTTCTTG	*sid2-2*
sid2-2R	TTACAAGAGAGACAACATTGCTTTC	
atchup1-LP2	CATCTAGCCAGCTAACGAACG	*chup1*
atchup1-RP2	CTTAAGAAATGGGGCAAAAGC	
LBb1.3	ATTTTGCCGATTTCGGAAC	*chup1, jac1*
atjac1-LP	ATGCCCTCTGGTACGATTTTC	*jac1*
atjac1-RP	TAGCCCGATACCCGATAAATC	
atcas-LP	CGCTTCTTTGATTACCAATCG	*cas*
atcas-RP	TCAAACCCTAAACCCCAAAAC	
LBb1	GCGTGGACCGCTTGCTGCAACT	*cas, phot1*
atphot1-LP	TCGAACATTTCTTTGCAAATTC	*phot1*
atphot1-RP	TCATCCAAAGATTCGCTCTTC	
LBa1	TGGTTCACGTAGTGGGCCATCG	*phot2*
atphot2-LP	TCCATCTCCTTTGAATGATGC	
atphot2-RP	AGTGTCATTGCTCACGGATTC	

Supplementary Table 3. Primers used for the RT-qPCR analysis.

**Name**	**Sequence (5'-3')**	**Target gene**
PAD3f	TGCTCCCAAGACAGACAATG	*PAD3*
PAD3r	GTTTTGGATCACGACCCATC	
CYP79B2f	AGGCAACCCATTGCTTACCGCC	*CYP79B2*
CYP79B2r	CCATTGCTTTACGGAGAATCTC	
CYP71A13f	TAAAGAGGTGCTTCGGTTGC	*CYP71A13*
CYP71A13r	TATCGCAGTGTCTCGTTGGA	
MYB51f	ACAAATGGTCTGCTATAGCT	*MYB51*
MYB51r	CTTGTGTGTAACTGGATCAA	
PDF1.2af	TTTGCTGCTTTCGACGCAC	*PDF1.2a*
PDF1.2ar	CGCAAACCCCTGACCATG	
PR1f	GGTAGCGGTGACTTGTCTGG	*PR1*
PR1r	CAAACTCCATTGCACGTGTT	
FRK1f	ACGGGCATAGTTCCACAAAG	*FRK1*
FRK1r	CGTCAAAAGAACGACGATGA	
NHL10f	TTCCTGTCCGTAACCCAAAC	*NHL10*
NHL10r	CCCTCGTAGTAGGCATGAGC	
