# Supplementary material for: Epidermal chloroplasts are defense-related motile organelles equipped with plant immune components
Source: Nat Commun. 2021 May 20;12:2739. doi: 10.1038/s41467-021-22977-5 (PMC8137707; doi:10.1038/s41467-021-22977-5)
Supplement: Supplementary file 3 — Descriptions of Additional Supplementary Files [file 41467_2021_22977_MOESM3_ESM.pdf]

Descriptions of Additional Supplementary Files

Supplementary Movie 1
Description: Intracellular movement of chloroplast in ECR-activated epidermal cell. Corb was inoculated onto pen2-1 plants, and the ECR was observed at 2 dpi. Epidermal chloroplasts were visualized using chlorophyll autofluorescence. Related to Supplementary Fig. 7.

Supplementary Movie 2
Description: 3D rotation projection of Z-stack images of epidermal and mesophyll chloroplasts with fluorescently labeled immune component GSH1. 3D rotation movie of Z-stacks of the gsh1-1 plants expressing GSH1::GSH1-GFP with DW treatment. Related to Supplementary Fig. 8.

Supplementary Movie 3
Description: 3D rotation projection of Z-stack images of epidermal and mesophyll chloroplasts with fluorescently labeled immune component GSH1. 3D rotation movie of Z-stacks of the gsh1-1 plants expressing GSH1::GSH1-GFP with Cfio inoculation. Related to Supplementary Fig. 8.

Supplementary Movie 4
Description: 3D rotation projection of Z-stack images of epidermal and mesophyll chloroplasts with fluorescently labeled immune component EDS5. 3D rotation movie of Z-stacks of the Col-0 plants expressing CaMV 35S::EDS5-sfGFP with DW treatment Related to Supplementary Fig. 8.

Supplementary Movie 5
Description: 3D rotation projection of Z-stack images of epidermal and mesophyll chloroplasts with fluorescently labeled immune component EDS5. 3D rotation movie of Z-stacks of the Col-0 plants expressing CaMV 35S::EDS5-sfGFP with Cfio inoculation. Related to Supplementary Fig. 8.

Supplementary Movie 6
Description: 3D rotation projection of Z-stack images of epidermal and mesophyll chloroplasts with fluorescently labeled immune component CAS. 3D rotation movie of Z-stacks of the Col-0 plants expressing CaMV 35S::CAS-sfGFP with DW treatment. Related to Supplementary Fig. 8.

Supplementary Movie 7
Description: 3D rotation projection of Z-stack images of epidermal and mesophyll chloroplasts with fluorescently labeled immune component CAS. 3D rotation movie of Z-stacks of the Col-0 plants expressing CaMV 35S::CAS-sfGFP with Cfio inoculation. Related to Supplementary Fig. 8.

Supplementary Movie 8
Description: Changes in intracellular locations of fluorescently-labeled GSH1 protein with epidermal chloroplasts in ECR-activated epidermal cell. Cfio was inoculated onto gsh1-1 plants expressing GSH1::GSH1-GFP, and fluorescent signals of GSH1-GFP and autofluorescence of epidermal chloroplasts were simultaneously observed at 2 dpi.
